# Influence of Preoperative Radiation Therapy on the Occurrence of Pharyngocutaneous Fistula After Total Laryngectomy

**DOI:** 10.7759/cureus.13797

**Published:** 2021-03-10

**Authors:** Masaaki Higashino, Teruhito Aihara, Tetsuya Terada, Ryo Kawata

**Affiliations:** 1 Otolaryngology - Head and Neck Surgery, Osaka Medical College, Takatsuki, JPN

**Keywords:** laryngeal carcinoma, total laryngectomy, pharyngocutaneous fistula, radiation therapy, neck dissection, preoperative lymphocyte

## Abstract

Introduction

Chemo-radiotherapy (CRT) has increasingly been employed for the treatment of laryngeal cancer at T3 or higher rather than total laryngectomy (TL), in order to preserve the larynx. However, TL is still frequently performed in patients with advanced laryngeal cancer, especially T4 disease. When CRT is performed for advanced cancer, there is a certain rate of residual disease or relapse, and TL is conducted as salvage surgery for those patients, but TL following CRT is associated with a high incidence of postoperative complications.

Objective

The purpose of this study was to investigate the influence of preoperative radiation therapy on the occurrence of postoperative complications of TL, particularly pharyngocutaneous fistula (PCF).

Methods

We retrospectively investigated 142 patients who underwent TL for laryngeal cancer whether postoperative complications were related to a history of radiation therapy or neck dissection. Detailed investigation of the 32 patients who underwent radiation therapy was also conducted.

Results

PCF was significantly higher after radiation therapy. Neck dissection was not related. As the time from radiation therapy to TL decreased, the incidence rate of postoperative PCF increased and the time to closure became significantly longer. Preoperative laboratory tests did not show a significant difference in Hb and Alb, but the lymphocyte count was significantly lower in patients with PCF.

## Introduction

Recently, chemo-radiotherapy (CRT) has increasingly been employed for the treatment of laryngeal cancer at T3 or higher rather than total laryngectomy (TL), in order to preserve the larynx [1-4]. However, TL is still frequently performed in patients with advanced laryngeal cancer, especially T4 disease. When CRT is performed for advanced cancer, there is a certain rate of residual disease or relapse, and TL is conducted as salvage surgery for those patients, but TL following CRT is associated with a high incidence of postoperative complications [5-6].

Concerning the complications of TL, Ganly et al. [7]. reported that the major complications are pharyngocutaneous fistulas (PCFs) requiring local intervention or surgical closure, extensive skin necrosis, and large vessel rupture while the minor complications are small salivary fistulas (detected by diagnostic imaging), minor skin necrosis, and peri-tracheostomy inflammation/necrosis. If major complications occur, swallowing is severely affected and quality of life (QOL) shows marked impairment. Moreover, admission is often needed for interventions, so hospitalization can become protracted. PCF not only prolongs hospital stay but also requires daily wound care, leading to a significant decline in patient QOL. Occasionally, the infection spreads from the PCF, disrupting blood vessels and leading to fatal conditions. Therefore, efforts should be made to avoid PCFs as much as possible.

We reviewed the postoperative complications of patients who underwent TL at our hospital, and we investigated the influence of preoperative radiation therapy on the occurrence of complications, particularly PCF.

## Materials and methods

Patients

This study retrospectively investigated 142 patients who underwent TL for laryngeal carcinoma at the department of otolaryngology and the department of head and neck surgery at Osaka Medical College from 1999 through 2018. There were 138 male patients and four female patients with a median age of 71 years (range: 45-90 years). The primary sites of the larynx were the supraglottic in 85 patients, the glottic in 56 patients, and the subglottic in one patient. Among the 142 patients, 32 patients were recurrence carcinoma who received radiation therapy previously. They were all men with a median age of 68 years (range: 45-87 years). Four patients underwent concurrent neck dissection while 28 patients did not. The median preoperative dose of radiation was 70 Gy (range: 45-75 Gy). Twenty-two patients only received radiation therapy and 10 patients received CRT. The target region was the entire neck in six patients and was localized to the larynx in 26 patients.

Methods

In the 142 patients who underwent TL, the incidence of major and minor complications was determined. After total laryngectomy, fluoroscopy with barium was performed 10 to 14 days after surgery with or without RT. The absence of a leak was confirmed, and oral intake was started. Major complications were defined as pharyngocutaneous fistulas (PCF) requiring local intervention or surgical closure, extensive skin necrosis, and large vessel rupture while minor complications were defined as small salary fistulas (detected by diagnostic imaging), minor skin necrosis, and peri-tracheostomy inflammation/necrosis. In addition, we investigated whether postoperative complications and a history of radiation therapy or neck dissection were related. Detailed investigation of the 32 patients who underwent radiation therapy was also conducted. Among patients receiving radiation therapy, the major complication was always PCF. Patients with and without PCF were categorized as Group A (n=7) and Group B (n=25), respectively, for comparison of the following parameters: diabetes, body mass index (BMI), preoperative laboratory parameters (albumin, hemoglobin, and lymphocyte count), preoperative radiation dose and target area, concurrent chemotherapy administered with radiation therapy, and the time from radiation therapy to salvage surgery. We also compared the timing of development of PCF, the time from the occurrence of PCF to closure, and the outcome between Group A and five other patients with PCF who did not receive preoperative radiation therapy (Group C). The study protocol was approved by the institutional review board at Osaka Medical College (2032-1).

Statistical analysis

The Mann-Whitney U-test, chi-square test, or Fisher’s exact test was performed to assess statistical significance while the Kaplan-Meier method was used to compare the local control rate. The level of significance was set at p<0.05.

## Results

Results

1. Postoperative Complications After TL

Among all 142 patients, major complications occurred in 13 patients (9.2%) and minor complications occurred in 11 patients (7.7%). The major complication was PCF in 12 patients and extensive skin necrosis in one patient. Minor complications were a small salivary fistula detected on diagnostic imaging in four patients, minor skin necrosis in four patients, a subcutaneous abscess in two patients, and a small hematoma in one patient.

**Figure 1:**
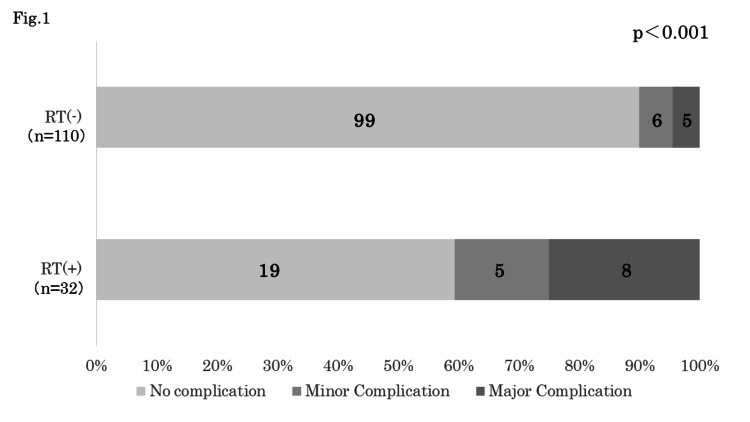
Postoperative complications after TL and the history of radiation therapy The incidence of postoperative complications was significantly higher in patients who received radiation therapy than patients without radiation therapy (p<0.001). TL: total laryngectomy

Among the 110 patients without radiation therapy, 11 patients experienced postoperative complications, with major complications being observed in five patients (4.5%) and minor complications in six patients (5.5%). Among the 32 patients who received radiation therapy, postoperative complications were observed in 13 patients, with major complications being observed in eight patients (25%) and minor complications in five patients (15.6%). The incidence of postoperative complications was significantly higher in the latter group (p<0.001) (Figure 1).

**Figure 2:**
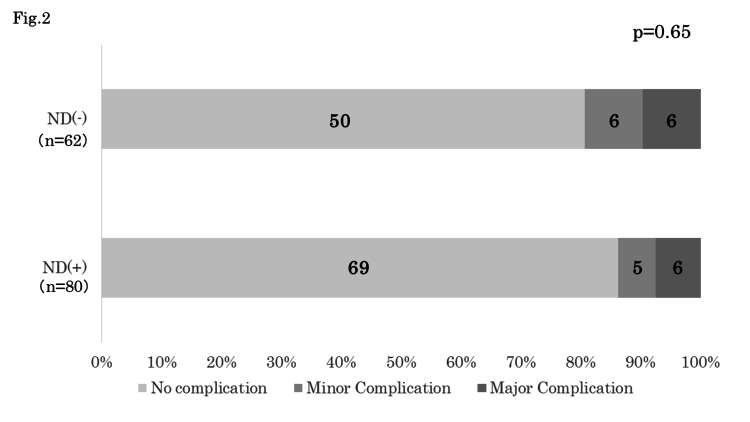
Postoperative complications after TL and the neck dissection There was no significant difference in the frequency of complications whether conducted neck dissection was conducted or not (p=0.65). TL: total laryngectomy

Neck dissection was conducted in 80 patients undergoing TL while 62 patients did not receive neck dissection (including patients with Level VI dissection only). Among the 80 patients with neck dissection, 11 patients experienced postoperative complications, which were major in six patients (7.5%) and minor in five patients (6.3%). Among the 62 patients without neck dissection, 12 patients experienced postoperative complications, which were major and minor complications in six patients each (9.7%). There was no significant difference in the frequency of complications between these two groups (p=0.65) (Figure 2).

The incidence of PCF was investigated in relation to a history of radiation therapy and neck dissection. Among 32 patients with a history of radiation therapy, four patients received neck dissection and PCF occurred in two of them (50%). Among the 28 patients without neck dissection, PCF occurred in five patients (17.9%). On the other hand, among the patients without a history of radiation therapy, PCF occurred in four (5.3%) patients among the 76 patients with neck dissection and one patient (2.9%) among 34 patients without neck dissection (Table 1).

**Table 1:** Relationship between radiation therapy and neck dissection on PCF Concurrent neck dissection tended to induce PCF in patients with a history of radiation therapy, and patients with an unfavorable outcome of PCF had both preoperative radiation therapy and neck dissection. PCF: pharyngocutaneous fistula

	RT(+)	RT(-)	Total
ND(+)	2／4	4／76	6／80
ND(-)	5／28	1／34	6／62
Total	7／32	5／110	12／142

2. Complications in 32 Patients With Radiation Therapy (Table 2)

**Table 2:** Complications in 32 patients with radiation therapy Patients with and without PCF were categorized as Group A (n=7) and Group B (n=25), respectively, for comparison of the following parameters: diabetes, BMI, preoperative laboratory parameters (albumin, hemoglobin, and lymphocyte count), preoperative radiation dose and target area, concurrent chemotherapy administered with radiation therapy, and the time from radiation therapy to salvage surgery. PCF: pharyngocutaneous fistula; BMI: body mass index; RT: radiotherapy; CRT: chemo-radiotherapy

	Group A	Group B	p-value
Sex (Men: Women)	7 : 0	25 : 0	‐
Median age	67 (61-83）	70 (45-87）	0.63
Diabetes mellitus	2 : 5	4 : 21	0.84
BMI	22.3 (18.2-27.2）	20.0 (16.3-28.4）	0.34
Preoperative albumin	4.1 (3.5-4.7)	4.1 (3.2-5.2)	0.60
Preoperative hemoglobin	13.7 (11.4-16.7)	13.7 (10.1-15.5)	0.78
Preoperative peripheral blood lymphocyte	864 (407-1909)	1275 (600-2573)	0.029
Radiation dose (Gy)	70 (64-75)	70 (45-70)	0.56
Radiation field (larynx : total neck)	4 : 3	22 : 3	0.19
RT : CRT	3 : 4	19 : 6	0.23

The seven patients in Group A with PCF and 20 patients in Group B were all men. The median age was 67 years (range: 61-83 years) in Group A and 70 years (45-87 years) in Group B (p=0.84). The tumor sites were supraglottic, glottic, and subglottic in two, four, and one patient from Group A versus eight, 16, and one patient from Group B, respectively.

Six of the 32 patients had diabetes, including two patients (28.6%) of seven patients from Group A and four patients (16%) of 25 patients from Group B. There was no significant difference in the frequency of diabetes between the two groups (p=0.84).

The median preoperative BMI was 22.3 kg/m^2^ (range: 18.2-27.2) in Group A and 20.0 kg/m^2^ (16.3-28.4) in Group B, showing no significant difference (p=0.34). The median preoperative serum albumin level was 4.1 g/dl (3.5-4.7) in Group A and 4.1 g/dl (3.2-5.2) in Group B, and it was not significantly different (p=0.60). The median preoperative hemoglobin was 13.7 g/dl (11.4-16.7) in Group A and 13.7 g/dl (10.1-15.5) in Group B, also showing no significant difference (p=0.78). Finally, the median preoperative lymphocyte count was 864/mm^3^ (407-1909) in Group A and 1275/mm^3^ (600-2573) in Group B, being significantly lower in Group A than Group B (p=0.029).

The median preoperative radiation dose was 70 Gy (64-75 Gy) in Group A and 70 Gy (45-70 Gy) in Group B, with no significant difference (p=0.56). With regard to the target region of preoperative radiation, it was localized to the larynx or was the entire neck in four and three patients from Group A versus 22 and three patients from Group B, respectively, with no significant difference (p=0.19). Radiation therapy alone or CRT was performed in three and four patients from Group A versus 19 and six patients from Group B, showing no significant difference of treatment modalities (p=0.23).

**Figure 3:**
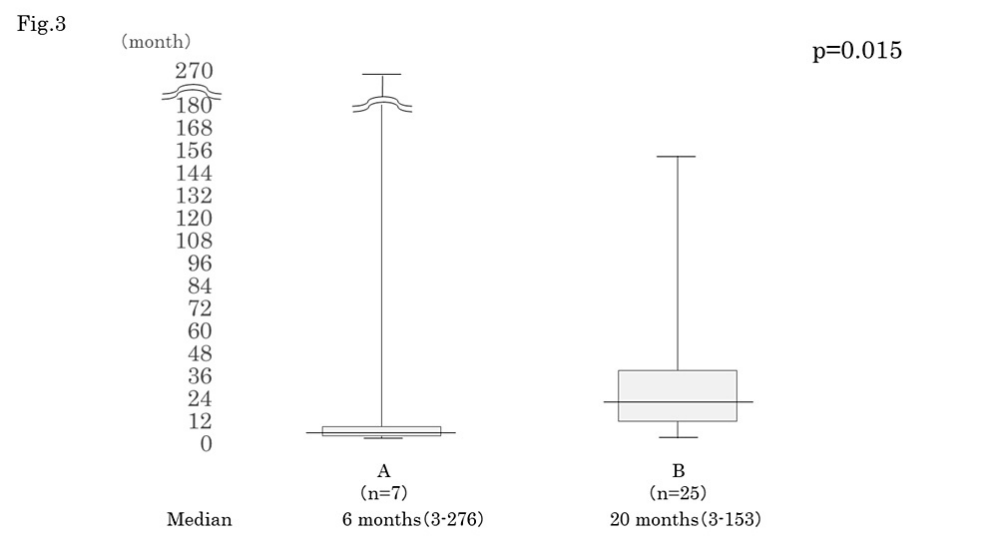
The interval from completion of radiation to salvage surgery (TL) The median interval from completion of radiation to salvage surgery (TL) was six months (3-276 months) in Group A and 20 months (3-153 months) in Group B, and it was significantly shorter in Group A than in Group B (p=0.015). TL: total laryngectomy

The median interval from the completion of radiation to salvage surgery (TL) was six months (3-276 months) in Group A and 20 months (3-153 months) in Group B, and it was significantly shorter in Group A than in Group B (p=0.015) (Figure 3).

3. Timing of PCF and Clinical Course of PCF Patients (Table 3)

**Table 3:** Timing of PCF and clinical course of PCF patients The clinical course of seven patients with PCF and a history of radiation therapy (Group A) was compared with that of five patients without a history of radiation therapy who also developed PCF (Group C). PCF: pharyngocutaneous fistula; LDMC: latissimus dorsi musculocutaneous; PMMC: pectoralis major myocutaneous

	Radiation therapy	Neck dissection	Postoperative PCF occurred (days）	Method for close PCF	Days until PCF closed
①	＋	－	3	Local treatment	57
②	＋	－	11	Anterior chest flap	80
③	＋	－	12	Local treatment	93
④	＋	－	14	LDMC, PMMC flap	106
⑤	＋	－	20	PMMC flap	120
⑥	＋	Unilateral	19	Death because of sepsis (60)	Not closed
⑦	＋	Bilateral	21	Death for raptured carotid artery (56)	Not closed
⑧	－	－	5	Local treatment	23
⑨	－	－	8	Local treatment	14
⑩	－	Bilateral	7	Hinge flap	69
⑪	－	Bilateral	10	Anterior chest flap	78
⑫	－	Bilateral	15	Local treatment	61

The clinical course of seven patients with PCF and a history of radiation therapy (Group A) was compared with that of five patients without a history of radiation therapy who also developed PCF (Group C). The median time to the occurrence of PCF was 14 days (3-21 days) in Group A and eight days (5-15 days) in Group C, which was not significantly different (p=0.14). Concerning PCF surgery, closure was achieved by local intervention in two patients from Group A. However, pedicle flaps were needed for closure in three patients, including an anterior chest skin flap in one patient, a latissimus dorsi musculocutaneous (LDMC) flap + pectoralis major musculocutaneous (PMMC) flap in one patient, and a PMMC flap in one patient. For the remaining two patients, surgery could not achieve closure and the outcome was unfavorable (one patient died of septicemia on Day 60 and the other patient died of cervical arterial rupture on Day 56). Among the five patients in Group C, closure of PCF was achieved in three patients by local intervention, a hinge flap was used in one patient, and one patient required a pedicle flap (anterior chest skin flap). The median time from the occurrence of PCF to closure was 106 days (57-120 days) in Group A and 65 days (14-78 days ) in Group C, being significantly longer in Group A (p=0.047). There were no significant differences in diabetes, BMI, and laboratory test data between the two groups.

## Discussion

With regard to the complications of TL, it has been reported that the incidence of PCF is 7.4%-58% and that it occurs eight to 13 days postoperatively. The duration of hospitalization is significantly prolonged if PCF occurs [8-10]. In the present study, PCF occurred in 12 out of 142 patients (8.5%) and the median time of occurrence was 12 days (3-21 days) postoperatively. Among the 12 patients, PCF occurred in five out of 110 patients (3.6%) without a history of radiation therapy versus seven out of 32 patients (21.9%) with a history of radiation therapy. Thus, the incidence of PCF was higher after radiation therapy. In addition, as the time from radiation therapy to TL decreased, the incidence rate of postoperative PCF increased and the time to closure became significantly longer. There was no relationship of PCF with the tumor site. Preoperative laboratory tests did not show a significant difference in hemoglobin (Hb) and albumin (Alb), but the lymphocyte count was significantly lower in patients with PCF. It was previously reported that delivery of 65-70 Gy of radiation therapy alone to head and neck cancer did not reduce the white blood cell (WBC) count, but the lymphocyte count was reduced by about 30% versus the pretreatment value and about 60 months was required for recovery. Accordingly, the immune function of patients may be impaired for a certain period after radiation therapy to the head and neck [11-12]. Recently, rather than radiation therapy alone, CRT has been performed as the more potent treatment for laryngeal preservation. However, the impact of CRT on bone marrow is considered to be more severe than that of radiation therapy alone. In other words, in patients who experienced relapse shortly after radiation therapy, the peripheral lymphocyte count was reduced and immunity was impaired, so there was a higher risk of wound infection following TL. Thus, the risk of developing PCF may also have been increased. Therefore, the initial curative use of (C)RT needs to be considered carefully. It seems that using (C)RT just to preserve the larynx should be avoided in patients with advanced cancer since the risk of residual tumor is high. If PCF develops after total laryngectomy, reoperation before closure may be required after long-term local treatment. Most patients, especially those with a history of radiation, require a closure period of at least two months, and gastrostomy should be considered immediately for nutritional support.

Radiation therapy has various adverse effects on tissues. Epithelial/parenchymal lesions cause atrophy, necrosis, cellular atypia, and interstitial fibrosis and necrosis. It also causes fatal and sublethal damage to endothelial cells in blood vessels with capillary rupture or thrombosis. They result in delayed wound healing [13]. Surgery performed after radiation therapy is associated with an increase in the risk and severity of complications. We found that the preoperative radiation dose and concurrent chemotherapy did not affect the onset of PCF. However, the incidence of PCF was higher in patients with full neck radiation exposure than patients with radiation restricted to the larynx. Moreover, concurrent neck dissection tended to induce PCF in patients with a history of radiation therapy, and the patients with an unfavorable outcome of PCF had both preoperative radiation therapy and neck dissection. This suggests that inflammation may easily extend to the lateral neck region once infection occurs in such patients. Therefore, prophylactic neck dissection should be performed with caution in patients with a history of preoperative radiation therapy. We perform prophylactic neck dissection when TL is conducted as part of initial treatment, specifically for T3-4N0 supra-glottal cancer. However, when TL is done for relapse after radiation therapy, we do not perform prophylactic neck dissection due to the high risk of postoperative complications.

Curative treatment of laryngeal cancer with (C)RT is very difficult because the risk of relapse is increased if the dose is insufficient, while an excessive radiation dose increases the risk of functional impairment. In our series, concurrent chemotherapy and radiation therapy improved the local control rate in patients with T2N0 glottic cancer. However, the risk of laryngeal dysfunction, such as vocal cord paralysis and laryngeal necrosis, was reported to be higher with (C)RT [14]. Our study also indicated that when surgery was performed following (C)RT and the tissues were weakened, wound healing was negatively affected and PCF was more likely to occur due to increased susceptibility to infection. This may have been due to impairment of immune function resulting from a decrease of peripheral lymphocytes following radiation exposure. Therefore, the indiscriminate use of (C)RT should be avoided for locally advanced laryngopharyngeal cancer with a risk of early relapse. Finally, when selecting (C)RT as the initial therapy for advanced laryngopharyngeal cancer, the patient should be well-informed about the risks of TL as salvage surgery.

## Conclusions

The indiscriminate use of radiation therapy should be avoided for locally advanced laryngopharyngeal cancer with a risk of early relapse. When selecting CRT for laryngeal preservation for locally advanced laryngeal cancer, adequate informed consent is required, including various risks at relapse.
